# Prominent Fatigue but No Motor Fatigability in Non-Hospitalized Patients With Post-COVID-Syndrome

**DOI:** 10.3389/fneur.2022.902502

**Published:** 2022-07-01

**Authors:** Christian Weich, Christian Dettmers, Romina Saile, Luise Schleicher, Manfred Vieten, Michael Joebges

**Affiliations:** ^1^Department of Sports Science, University of Konstanz, Konstanz, Germany; ^2^Kliniken Schmieder, Konstanz, Germany

**Keywords:** post-COVID-syndrome, gait analysis, attractor method, motor fatigability, motor fatigue

## Abstract

**Objectives:**

Fatigue is a frequent and often disabling symptom in patients with post-COVID syndrome. To better understand and evaluate the symptom of motor fatigue in the context of the post-COVID syndrome, we conducted treadmill walking tests to detect the phenomenon of motor fatigability or to evaluate whether evidence of organic lesions of the motor system could be found, similar to patients with multiple sclerosis.

**Method:**

Twenty-nine non-hospitalized patients with post-COVID syndrome completed the Fatigue Scale for Motor and Cognitive Function (FSMC) questionnaire to determine the trait component of subjective fatigue before they were tested on a treadmill walking at a moderate speed for up to 60 min or until exhaustion. During the walking test oxygen uptake, ventilation and acceleration data of both feet were collected. To determine motor performance fatigability, the Fatigue Index Kliniken Schmieder (FKS) was calculated using the attractor method.

**Results:**

The average walking duration was 42.7 ± 18.6 min with 15 subjects stopping the walking test prematurely. The FSMC score revealed a severe cognitive (37.6 ± 8.2) and motor (37.1 ± 7.8) fatigue averaged over all subjects but only two subjects showed an FKS above the normal range (>4), representing performance fatigability. There was no significant correlation between subjective fatigue (FSMC) and FKS as well as walking time. Absolute values of oxygen uptake and ventilation were in the normal range reported in literature (*r* = 0.9, *p* < 0.05), although eight subjects did not produce a steady-state behavior.

**Conclusion:**

Almost all patients with post-COVID syndrome and subjectively severe motor fatigue, did not show motor fatigability nor severe metabolic anomalies. This is argued against organic, permanent damage to the motor system, as is often seen in MS. Many of the patients were - to our and their own surprise - motorically more exertable than expected.

## Introduction

While in the beginning, the Severe Acute Respiratory Syndrome Coronavirus 2 (SARS-CoV-2) was thought to be a viral airway infection, lasting rarely longer than 14 days in mild cases, it is now realized that a considerable number of patients have long-lasting symptoms (1, 2). Post-COVID syndrome has arrived in the mainstream of medicine and is challenging the health system (3). Many patients were advised during the acute phase to stay at home in quarantine and not to visit their general practitioner. Many patients felt left alone with their disease and in the first few months of 2020, symptoms of post-COVID have been primarily described, exchanged, and advocated in patients' forums and social media. This encouraged publications stating that post-COVID is the first patient-made disease (4). Symptoms have been described and empathically shared on social media. This might have contributed to the fact that post-COVID syndrome has been taken up by scientists and later by health professionals and politicians for debate (5).

The first publications focused on the symptomatology, with most often cited symptoms of fatigue, myalgia, dyspnea, headache, sleep disturbances, cognitive disturbances (“brain fog”), and post-exertional fatigue (6). Symptomatology seems to be independent of the seriousness of the primary infection (1, 2). Patients are often on sick leave for months, and some of them have difficulties returning to work (3). Six months after the primary infection, an online survey showed that 22% were still not working and 45% required a reduced working schedule (7). Prevalence rates of symptoms and time course during the first 7 months have been elaborately described (7). The precise pathophysiology remains poorly understood (8). There seems to be no correlation between the degree of symptoms and biomarkers [like CRP, interleukin, etc. (6)], albeit there is evidence that they are linked to chronic (possibly autoimmune) inflammations (9). While publications on symptoms and prevalence rates grow rapidly, the precise etiology in individual patients and in single case studies and the contributing psychosocial risk factors remain obscure.

A different disease, in which fatigue is very prominent and often the most devastating symptom, is multiple sclerosis (MS). In the field of MS, discrimination between fatigue and fatigability has been introduced (10) and has been shown to be extremely helpful (11). Fatigue represents the subjective sensation of the patient, while fatigability represents the change in performance, which can be measured (10). In addition, state fatigue represents the short-lasting, momentary condition often depicted by a visual analog scale. Trait fatigue reflects a long-lasting condition, often regarding the last 4 weeks. It is most often captured in one of the many fatigue scales (12). Besides motor and cognitive fatigue, there is a third category termed emotional (or psychosocial) fatigue (13).

The advantage of the new terminology is that fatigability can be measured and observed. Many patients with MS, who suffer from motor fatigability, show increasing weakness during exhaustion, for instance, increasing foot drop or proximal weakness, which might also cause increasing spasticity or ataxia. If it is very prominent, the neurologist can observe motor fatigability by comparing the gait of the exhausted patient with his normal gait. More sophisticated are measurements using motion-sensitive (IMU) sensors fixed to the ankle in combination with an attractor-based evaluation (14–16). This change in gait performance is not found in, e.g., depressive disorders (17) and is interpreted as a demonstration of an organic lesion of the central nervous system, possibly comparable to a use-or activity-dependent conduction block (18). The correct discrimination between organic and psychological causes of fatigue and fatigability is helpful to define the best therapy in individual cases of MS.

Besides a sophisticated gait analysis, we also investigated oxygen uptake and ventilatory data to document an adequate load for the treadmill test for each individual patient. At the same time, these parameters allow the identification of a potential insufficient ventilatory capacity as a potential consequence of SARS-CoV-2 infection. Oxygen uptake during submaximal continuous exercise will initially increase monoexponentially from a resting state (~3.5 ml/min/kg) until it finally settles in a steady state (after ~3 min) when exercising at a constant, moderate load (walking speed) (19). The resulting intensity was proven to be suitable for gait exercises in a rehabilitative context (20). Normal values for oxygen consumption for a constant walking load on a treadmill for a common range of 3–6 km/h correspond to about 8–18 ml/min/kg when at a steady-state load (21). This corresponds comparably with minute ventilation (L/min), which facilitates an increased oxygen exchange. At light intensities, as defined above, a turnover of 25–40 L/min can be expected as the increase is predominantly due to adjustments to the tidal volume (22).

Motor fatigue is a very prominent finding in patients with post-COVID syndrome (7). The aim of our study was a first attempt to disentangle organic components of motor fatigue. We did not imply that we can rule out other potential phenomena like endothelial, mitochondrial, or other dysfunctions. In early 2021, our first approach was to exclude one potential mechanism of organic dysfunction knowing that this might not be the only or last option. To be more specific, the intention was to investigate whether we can identify changes in gait pattern, oxygen consumption, and ventilation during physical exertion as indications of an organic failure. We evaluated rather mildly affected patients – none of them had been hospitalized during the acute infection – suffering from motor fatigue after being infected with SARS-CoV-2 in an exertional test on a treadmill.

## Materials and Methods

### Patient Demographics and Medical History

The inclusion criteria of our study were the diagnosis of SARS-CoV-2 (proven or suspected), initially non-hospitalized post-COVID syndrome, subjective fatigue, being able to walk on a treadmill without holding the side rails, and sick leave before admission for several months. A total of 29 patients with post-COVID syndrome, 24 women and five men, were included in the study between May 2021 and February 2022 during their rehabilitation at the Kliniken Schmieder Konstanz (Germany). Patients were aged 47.6 (± 10.02) years and weighed 80 (± 18.92) kg. Most of our patients had a thorough cardiac and pulmonary investigation without any pathological finding, which might have explained their symptoms or sick leave before they were referred to our rehabilitation clinic. The majority of our patients were able to dress and bath themselves. They could follow their training schedule and attend the sessions on their own and did not rely on support from nurses (Barthel Index >70). Most of them had been referred to a rehabilitation setting by the Berufsgenossenschaft (the organization responsible for diseases and accidents caused by or during work), by their pension fund, by their health insurance company, or by their neurologist or general practitioner due to protracted sick leave. All patients suffered from a SARS-CoV-2 infection during the first month of 2020 (first wave), during the end of 2020/beginning of 2021 (second wave), or during spring 2021 (third wave). Twenty-five patients had been tested with a polymerase chain reaction (PCR) test during the course of the disease. None had been hospitalized during the acute phase. All of them were on sick leave before being admitted to our clinic. The average duration of sick leave was 8.75 (± 6.2) months. Twelve patients had returned to work after the period of acute illness, and nine had deteriorated at some stage and were unable to continue their work. Six had been continuously on sick leave since their acute phase. The study was approved by the local ethical committee of the University of Konstanz (Germany) under the RefNo: 44/2021. All of the participants filled out and signed informed consent.

### Equipment

To acquire the raw data for the gait analyses, an inertial sensor (IMU) from RehaWatch (Magdeburg, Germany) was attached to each ankle of the patient with adhesive tape. The sensor was located directly above the lateral malleoli. Technically, the sensor works as a triaxial accelerometer with up to 16 G (1 G = 9.81 m/s^2^), a triaxial gyroscope with up to 2,000°/s, and a magnetometer. The raw data were collected with a sampling rate of 500 Hz with the corresponding RehaGait app (version 1.3.14; Hasomed, Magdeburg, Germany) to be saved internally for later use.

To conduct the ventilatory measurements, a mobile spirometry device (VO_2_Master, Vernon, Canada) with its associated app (version 0.22.10) was used. With this instrument (weight: 320 g; 200 g unit & 120 g mask), it was possible to determine the oxygen uptake as well as the ventilation breath-by-breath using a mask while walking. For all test sessions, the mouthpiece M, with a ventilation range of 15–180 L/min, was chosen. The walking tests were conducted on an HP COSMOS treadmill (model locomotion 150/50 DE med) equipped with a security harness.

### Study Design and Clinical Setting

**Figure 1:**
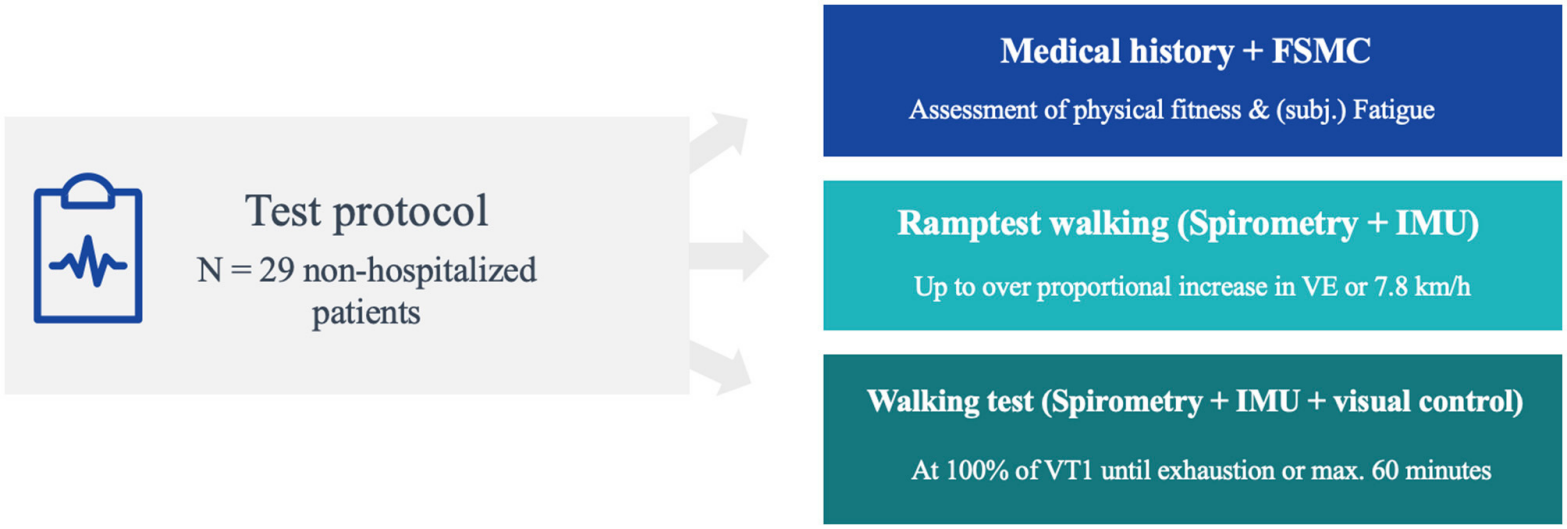
Overview of test protocol consisting of three parts: clinical interview, ramp test and walking test.

The study was undertaken as a prospective study in a neurological inpatient rehabilitation clinic. The clinic has the capacity for about 240 patients. The largest patient group encompasses patients with MS. The first patients with post-COVID-syndrome were admitted at the beginning of 2021. The therapeutic team consists of physiotherapists, occupational therapists, psychologists, speech pathologists, sports scientists, and vocational trainers. Patients stay for 4–5 weeks in the clinic. There is no acute neurological department, no intensive care, or early rehabilitation phase in our hospital. The test protocol was structured in three stages: First, all patients underwent a preliminary medical interview, followed by a ramp test, to determine the load level for the subsequent walking test, which had to be performed a few days later (see Figure 1).

#### Fatigue Scale (FSMC)

The Fatigue Scale for Motor and Cognition (FSMC) has been developed and introduced by Penner et al. (12). It is a questionnaire containing 20 items, which are answered on a Likert scale from one to five. The questionnaire is well evaluated compared to healthy people and is commonly used in patients with MS. Motor and cognitive fatigue can be separately evaluated; ranges are given for normal values (no fatigue), light motor and cognitive fatigue (>22 and >22, respectively), moderate motor and cognitive fatigue (>27 and >28), and severe motor and cognitive fatigue (>32 and >34). Values are also given for the overall fatigue (>43; >53; >63).

#### Ramptest

To determine an adequate exertion for the walking (performance fatigability) test, a ramp test on a treadmill was performed prior to a separate day. Once the participants were fixed with the security harness, they were allowed to familiarize themselves with the treadmill at a self-chosen speed for 5 min. Afterward, they started to walk at 1.0 km/h with 1% inclination for half a minute when the speed was increased by 0.3 km/h every further 30s. The walking test was either stopped as soon as the subjects claimed that they could not walk any further or when a maximum speed of 7.8 km/h was attained. Holding the side rails was not allowed to ensure free walking. The onset of physical activity from a resting condition is always linked to rapid and sensitive adaptations of the physiological systems. The increased energetic expenditure must be compensated accordingly. Sports science literature separates three intensity zones that can be determined spirometrically: regenerative, extensive, and intensive activity. The transitions from one intensity to another are defined by ventilatory threshold 1 (VT 1 or AerT) and ventilatory threshold 2 (VT2 or AnT) (20, 23). These thresholds can be detected by analyzing the respiratory gases and ventilation. VT1 correlates with the first lactate threshold, which is accompanied by the bicarbonate buffering of protons (H^+^). The increased release of carbon dioxide, also known as *excess CO*_2_, leads to an over-proportional increase in ventilation, which can be detected in the collected (breath-by-breath) spirometry data. Because this inflection point is not always easy to detect, a graphical determination is, even today, still preferred over full computational methods like the one provided by Orr et al. (24). The best known is certainly the V-Slope method (25), which draws regression lines, one in the lower and one in the steeper part of the ventilation-speed relationship. Once the best fit is determined relying on the *R*^2^ of each regression line, VT 1, the representative speed at VT 1 can be set at the intersection of both lines. The current test was always performed under medical supervision. Only subjects with spirometric data showing a clear VT1 were invited to participate in the performance fatigability test.

#### Continuous Walking Test (Performance Fatigability Test)

In order to adequately expose the patients to physical activity in the present study, an extensive continuous walking exercise was chosen to assess whether any motor fatigability symptoms or metabolic abnormalities occur. The test was undertaken within 1 week after the ramp test, with a walking speed set one step above where VT1 was detected (= VT1 + 0.3 km/h) and a treadmill inclination of 1%. By determining this workload level, the energy is predominantly derived from aerobic metabolism. This is to ensure that the intensity is sufficiently low so that a large number of metabolites (e.g., lactate) do not accumulate in the working muscles. It has been reported that this can promote a negative affective valence by increasing the effort perception (load-induced soreness), which can lead to an early termination of the test session (26). Subjects were instructed to walk until they felt they could no longer withstand the effort or for a maximal time of 60 min. To collect all motoric and metabolic raw data, the participants were equipped with an inertial measurement unit (IMU)-sensor attached to each ankle and a spirometer, as described earlier. The walking behavior was always visually observed, supported by a video recording from the back, and eventually, potential abnormalities, as well as the cause of termination, were written down. Spirometry and IMU data were collected throughout the entire session. The test was also performed under medical supervision. Since patients had been already familiarized with the treadmill in the first session (ramp test), the treadmill accelerated to the predetermined speed shortly after the start.

### Data Analysis

For the final assessment of fatigue, as well as performance fatigability and spirometric measures, the recorded raw data were further processed. FSMC scores were evaluated as described by Penner et al. (12). To interpret the behavior of respiratory variables, oxygen uptake (ml/min/kg) and minute ventilation (L/min) were evaluated. To provide an informative statement about motor patterns and gait behavior, algorithms of the attractor method (15) were applied.

#### Attractor Method (Kinematic Analysis)

The attractor method allows the analysis of human movements, especially cyclic motions like walking, running, cycling, or skiing. The approach was first described in 2013 (15) and is still being further developed to this day (16, 27–29). In this regard, it represents a feasible application in which handy IMU sensors can be used to capture motion data and evaluate it with respect to its development over time. In addition to applications in the context of sports (30–32), the attractor method has been established especially in rehabilitative diagnostics (14, 33–37). Attractors, a kind of average value of the covered gait cycles, are calculated minute by minute in order to subsequently rank them in relation to each other. In this way, modifications of two measured events can be evaluated not only with respect to the motion pattern itself (deltaM) but also concerning the motion accuracy (deltaD) (15, 38). In 2014, a specific application of these parameters, the so-called Fatigue Index Kliniken Schmieder (FKS or deltaF), was developed for the diagnosis of motor performance fatigability (14, 39). In the original methodology for determining the FKS (39), deltaM and deltaD were first calculated between the initial and final minute of a multi-minute walk test (for example the 6-min walking test) to finally multiply both values to deltaF. Recently, it was suggested to compare the last minute with the second minute, instead of the first, in order to obtain a more stable assessment (40). The so-called *transient effect* causes much larger oscillations at the beginning of a walking session, which eventually settle down after a short time (28). The established threshold for the occurrence of motor performance fatigability is deltaF ≥4 (39). In the present study, the FKS was determined as a parameter for motor performance fatigability for the continuous walking test, comparing the gait behavior of the last minute before termination and the second minute after the start of the test. For all fatigue indexes ≤4, the gait pattern can be assumed to be within the normal range.

#### Metabolic Assessment

Throughout the entire measurement, oxygen and ventilation data were collected via a portable spirometer (see above) on a breath-by-breath basis. First, an Augmented Dickey-Fuller test (41, 42) was performed for each data set (walking session) to check if it showed a clear steady-state signature. For each session, oxygen uptake and minute ventilation from the third minute after the start [respecting the initial *fast component* phase, see (19)] were analyzed. Subsequently, mean oxygen uptake (ml/min/kg) and ventilation (L/min) of the steady state [3 min until termination of effort] were determined for each case and checked with a paired sample *t*-test and a correlation analysis (IBM SPSS Version 28) if they are in accordance with literature-based norm values (21). In the context of our gait exertions, oxygen uptakes between 8–18 ml/min/kg (21) and minute ventilation of 20–40 L/min (22) can be expected (speed-dependent).

## Results

### Fatigue Scale for Motor and Cognition (FSMC)

The FSMC_Motor_, FSMC_Cognitive_, and FSMC_Total_ scores in 16 completely returned questionnaires averaged 37.1 ± 7.8 (= severe motor fatigue), 37.6 ± 8.2 (= severe cognitive fatigue), and 74.7 ± 14.9 (= severe total fatigue), respectively.

### Performance on the Treadmill

After assessing their ventilatory threshold (VT1), all rehabilitants participated in a motor performance fatigability test for a maximum of 60 min walking on a treadmill. The average walking time was 42.74 ± 18.6 min, with 14 patients reaching the full duration of 60 min. The shortest walking time was 9 min. Walking speed averaged 5.1 ± 1 km/h with a range of 1.9 to 6.4 km/h. The walked time (in min) of the fatigability test did not show a statistically significant correlation (*p* > 0.05) to subjective fatigue, operationalized using the FSMC [including only complete datasets (*n* = 16), see Fatigue Scale for Motor and Cognition (FSMC)].

### Metabolic Analyses

**Figure 2:**
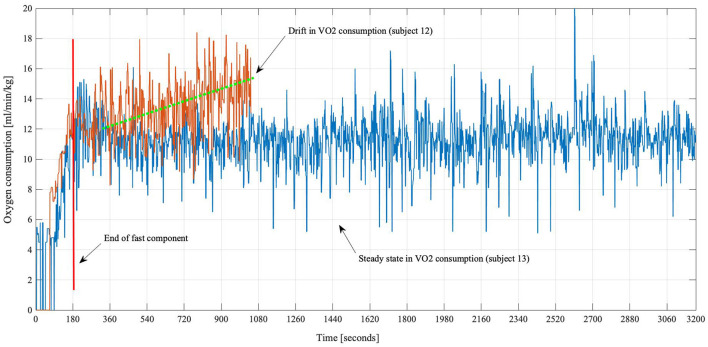
Oxygen consumption curves of two subjects. Subject 13 (blue) showed a normal oxygen kinetic, increasing from rest and leveling off until the walking session was stopped at the maximal time of 60 min. Subject 12 (orange) had to stop after approximately 17 min. It is visible that the VO_2_ consumption was steadily rising for subject 12.

Compared to an expected steady state of oxygen consumption and ventilation (see Figure 2, blue line), the results of the Augmented Dickey-Fuller tests revealed that eight rehabilitants out of 29 (27%) had a non-steady-state behavior (orange line) in both, their oxygen as well as ventilation, data (one example is shown in Figure 2). Looking solely at descriptive results, absolute values of oxygen uptake, in participants with a steady state, were 14.17 (± 3.2) ml/kg/min, and ventilation was 37.5 (± 7.8) L/min. For both, the statistical analyses showed that there is no significant difference (*p* = 0.16) as well as a high correlation (*r* = 0.9, *p* < 0.001) to the values provided in literature (21, 22).

### Fatigue Index Kliniken Schmieder (FKS)

**Figure 3:**
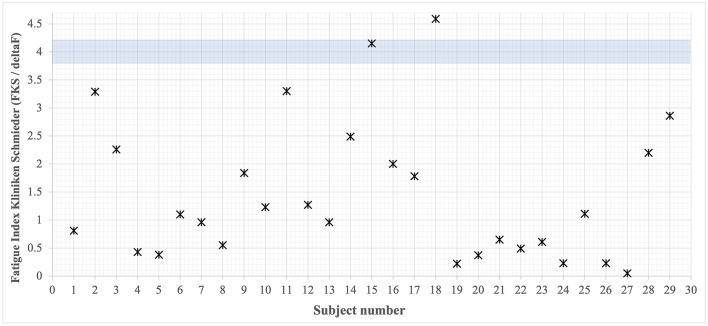
Overview of all rehabilitants' FKS. Cutoff value <4 [based on (42)] defines no present performance fatigability. Only two patients lay above this critical value.

The FKS is a sensitive measure to capture motor performance fatigability (17, 39). From all participating patients, attractors of their gait tests were calculated and the respective FKS was determined according to the attractor method algorithm (Figure 3). The findings show that all individuals, with only two exceptions (subjects 14 and 18), are well below the cutoff of FKS ≥4. Thus, motor performance fatigability cannot be attested in 94% of all cases. The attractor analysis (FKS in m/s^2^) during the fatigability test did not show a statistically significant correlation to subjective fatigue, operationalized using the FSMC [including only complete datasets (*n* = 16), see Fatigue Scale for Motor and Cognition (FSMC)].

## Discussion

The purpose of the present study was to investigate, whether changes in gait behavior, oxygen consumption, and ventilation during walking on a treadmill, as potential indications of an organic failure, can be observed. In all, except for two, initially non-hospitalized patients with post-COVID-syndrome complaining of serious motor fatigue [FSMC_Motor_ averaged 37.1 ± 7.8 (= severe Fatigue)], we found no gait abnormalities during walking till exhaustion; suggesting no organic lesion in the central motor system comparable to those found in patients with MS. The latter had also been reported in a previous study for patients with depression (17). Thus, this marks a striking difference from the common finding of motor fatigability in patients with MS (14, 39). We argue that this is an important outcome to begin to disentangle the complex, prominent and frequent phenomenon of fatigue in post-COVID syndrome. We were very much surprised that almost half of our patients were able to walk for 1 h on the treadmill at a fairly good speed around 5 km/h. Patients themselves were surprised that they managed to walk faster and longer than expected. Some of them complained about the backdrop the next day. Others did not experience the backdrop they had expected. From patients with MS, we know that fatigability while walking manifests at a particular localization in the nervous system where a focal infection caused a focal lesion which partially regenerated but left behind a “locus resistenciae minoris” – a weak point in the motor control system. When a patient is brought to his personal limit, such as walking at an unusually high speed, the latent lesion increases the weakness and causes a failure. Our study is in line with the hypothesis that a very careful neurological examination at rest might predict whether the patient will show fatigability during exertion or not. If there is no slight abnormality at rest, there might be no “locus resistance minoris” – no weak point, which gives way to fatigability during exertion. Finding no fatigability like that in MS does not exclude other potential organic causes like endothelial dysfunction, mitochondrial dysfunction, persisting inflammation, autoimmunity, or dysregulation of specific cytokines (6). It is the first step to disentangling the conundrum of post-COVID syndrome (8). Symptoms may also be related to psychosocial stress, trauma, or maladaptive coping style (43, 44). In contrast, common practice identification of an organic cause does not exclude psychological or psychiatric comorbidities and *vice versa*. In many neurological diseases, organic and psychogenic factors are both present and relevant and might exaggerate each other (45).

The described motor-related findings are also underlined by the fact that a normal oxygen and ventilation behavior was established in the majority of the patients. After the initial exercise-induced increase, a steady-state behavior was observed, which showed that these subjects were able to perform the specified exercise without respiratory difficulties. Exceptions (“drift”) from this behavior (see Figure 2, orange line) were only observed in persons who dropped out of the test markedly prematurely. Even though these data might be associated with the early dropout of the exercise, the absolute values of oxygen uptake and ventilation were within normal limits as it was demonstrated by the statistical tests compared to the literature. Also, based on the emotional expressions of early quitters, stress- or anxiety-related increase must be considered. In future studies, this could be assessed by questionnaires beforehand to determine the predisposition and afterwards to evaluate the actual occurrence of anxiety or negative expectations.

Describing what the phenomenon of fatigue in post-COVID is not like does not tell us what it is. However, it is the first step and the first approach to characterizing the complex phenomenon of fatigue. There is an obvious and broad discrepancy between the very frequent subjective complaints about fatigue and missing objective data of performance fatigability corroborating the complaints of the patients. The discrepancy between subjective complaints of the patients and missing objective deficits made some people claim that fatigue in post-COVID syndrome resembles or equals myeloencephalitis/chronic fatigue syndrome (ME/CFS). The subjective-objective cognitive mismatch in ME/CFS caused even a comparison to the functional cognitive disorder spectrum [(46) cited from (47)]. Fatigue, pain, and excessive interoceptive monitoring in ME/CFS may produce a shift from externally directed attention to subjective complaints, resulting in perceiving cognitive and motor tasks as extremely effortful (46).

Since there does not exist any motor, and only a few metabolic, potentially unspecific, anomalies emerging in our patient sample, we expect that psychosocial factors may be contributing or driving forces in the course of the disease for the patients. However, this can only be confirmed on the basis of future studies. The test design will be extended in order to allow a deeper investigation of the metabolic processes: Here, lactate measurements will be used as a marker for anaerobic energy utilization as well as heart rate data to gain insights into the acute response of the cardiovascular system. Furthermore, cooperative groups from the Schmieder Clinics as well as the University of Konstanz will conduct investigations on cognitive, emotional, and endocrine function or combined effects. Structured clinical interviews and neuroimaging (fMRI) will be used in our group to assess psychosomatic and psychiatric comorbidities.

We are optimistic that patients will not permanently suffer from fatigue when participating in adequate and intense exercise and cognitive behavioral training. Cognitive behavioral therapy will be a central component in our patient management besides individually tailored exercise and training. This might be even helpful in those patients in whom an organic trigger is identified. All of our patients were not initially hospitalized and represent a group of initially “mildly affected” patients. Thus, our observations and conclusions cannot be generalized to all post-COVID-19 patients and certainly not to those who had been ventilated in an intensive care unit. In those patients, one might often expect organic deficits, particularly concerning pulmonary and cardiac function or the central and peripheral nervous system.

## Conclusion

Initially, non-hospitalized patients with post-COVID syndrome should be examined with a holistic and multidisciplinary approach. After exclusion of cardiac and pulmonary deficits, patients in our sample with prominent fatigue did not show any signs of motor performance fatigability like patients with MS. This implies that we did not find signs of a lesion of the central motor system despite the prominent complaint of motor fatigue. It was not clear whether mild anomalies in ventilation were caused by metabolic or psychogenic alterations. Additional investigation of lactate and heart rate data will be helpful. Nevertheless, the test procedure used here has proven to be very useful for detecting motor and metabolic changes during physical exertion in patients complaining about fatigue. We assume that psychiatric and psychosomatic comorbidities may be involved in many initially non-hospitalized patients with post-COVID-syndrome.

## Data Availability Statement

The raw data supporting the conclusions of this article will be made available by the authors, without undue reservation.

## Ethics Statement

The studies involving human participants were reviewed and approved by University of Konstanz Ethics Committee (Ref No 44/2021). The patients/participants provided their written informed consent to participate in this study.

## Author Contributions

CW introduced important parts of the methodology and particularly the spirometric measurements and the ramp test. He collected and analyzed all data, wrote major parts of the manuscripts, and finalized the final version. CD initiated the study, supervised the treadmill test, and wrote an outline of the manuscript. RS organized and performed the ramp test and the exertional test. LS helped perform the treadmill test and collected data from the electronic patients' sheets. MV executed the attractor-based gait analysis and supervized the methodology of the gait analyses. MJ organized the study acquired patients, helped with the interpretation, and finalized the manuscript. All authors contributed to the article and approved the submitted version.

## Funding

CW and RS were partially supported by the Lurija Institute.

## Conflict of Interest

The authors declare that the research was conducted in the absence of any commercial or financial relationships that could be construed as a potential conflict of interest.

## Publisher's Note

All claims expressed in this article are solely those of the authors and do not necessarily represent those of their affiliated organizations, or those of the publisher, the editors and the reviewers. Any product that may be evaluated in this article, or claim that may be made by its manufacturer, is not guaranteed or endorsed by the publisher.
